# Infection with Seasonal H1N1 Influenza Results in Comparable Disease Kinetics and Host Immune Responses in Ferrets and Golden Syrian Hamsters

**DOI:** 10.3390/pathogens12050668

**Published:** 2023-04-30

**Authors:** Jemma Paterson, Kathryn A. Ryan, Daniel Morley, Nicola J. Jones, Paul Yeates, Yper Hall, Catherine J. Whittaker, Francisco J. Salguero, Anthony C. Marriott

**Affiliations:** UK Health Security Agency, Porton Down, Salisbury SP4 0JG, UK; jemma.paterson@ukhsa.gov.uk (J.P.); daniel.morley@ukhsa.gov.uk (D.M.);

**Keywords:** influenza, Golden Syrian hamster, ferret, animal models

## Abstract

Animal models of influenza are important in preclinical research for the study of influenza infection and the assessment of vaccines, drugs and therapeutics. Here, we show that Golden Syrian hamsters (*Mesocricetus auratus*) inoculated via the intranasal route with high dose of influenza H1N1 display comparable disease kinetics and immune responses to the ‘gold standard’ ferret (*Mustela furo*) model. We demonstrate that both the hamster and ferret models have measurable disease endpoints of weight loss, temperature change, viral shedding from the upper respiratory tract and increased lung pathology. We also characterised both the humoral and cellular immune responses to infection in both models. The comparability of these data supports the Golden Syrian hamster model being useful in preclinical evaluation studies to explore the efficacy of countermeasures against influenza.

## 1. Introduction

Influenza virus infection is an ongoing threat to global human health with illnesses ranging from mild to severe and hospitalisations and death occurring in high-risk groups. Annual seasonal epidemics are estimated to result in approximately 3 to 5 million cases of severe illness and up to 650,000 deaths [1]. Vaccination is the most effective way to prevent disease from influenza infection, however, due to antigenic drift influenza viruses continually evolving to escape existing immunity in the human population, necessitating annual vaccination, especially amongst high-risk groups. 

Ongoing assessment of novel vaccine and treatment strategies against influenza is key to preventing severe disease and death, and the preclinical evaluation of these countermeasures in animal models forms a critical part of this assessment. The gold standard animal model for human influenza research is the ferret due to their comparative symptomology, similar receptor distribution and the ability to be infected with human isolates without the need for adaptation [2]. Mice have also been extensively used to assess countermeasures against influenza but often require adaptation of clinical strains, limiting their use in human influenza virus research [2,3]. More recently, the Golden Syrian hamster has become an attractive small animal model for influenza virus research [4]. They are now a well-established model of influenza, although they are still used much less frequently than mice or ferrets.

Previously published data have demonstrated that selection of influenza strain is important for the Golden Syrian hamster, as some strains are so mild in the model that typical markers of infection such as weight loss and viral shedding from the upper respiratory tract are not detected [5]. Therefore, selecting strains that are able to produce robust disease endpoints following infection is important [6], and characterising the host immune response to infection is essential in order to optimise the model to effectively evaluate the immunogenicity and efficacy of countermeasures. 

Here, we demonstrate how the ferret and hamster models of influenza compare following infection with a human H1N1 virus strain, A/Guangdong-Maonan/SW1536/2019 (GM19). This strain was selected as a model of a contemporary circulating H1N1 that would be suitable to assess the effectiveness of countermeasures. We show that following infection, both models have measurable disease endpoints of weight loss, temperature change, viral shedding from the upper respiratory tract and increased lung pathology. The host responses to infection, both humoral and cellular, were also characterised in both models. The comparability of these data supports the Golden Syrian hamster model being useful in preclinical evaluation studies to explore the efficacy of vaccines, therapeutics and countermeasures against influenza.

## 2. Materials and Methods

**Viruses.** Influenza A/Guangdong-Maonan/SW1536/2019 (H1N1, EPI_ISL_419003) was propagated in SPF embryonated chicken eggs obtained from Charles River Ltd. (ECACC, Porton Down, UK). Virus titre was determined by plaque assay on MDCK cells under an agar overlay, followed by staining with crystal violet.

**Focus forming assay (FFA).** MDCK-SIAT cells were counted using Trypan Blue and a C-Chip haemocytometer and seeded in 96-well pates at 1.5 × 10^5^ cells/mL (1.5 × 10^4^ cells/well). Plates were incubated overnight at 37 °C, 5% CO_2_ in a humidified incubator. Samples were added neat with subsequent 1 in 10 serial dilutions in serum-free media (DMEM) to 10^−5^. A negative control (media only) and a positive control (A/California/07/2009) were included on each plate. After overnight incubation, the cell plates were washed twice with 200 µL PBS per well, and a 100 µL sample from the dilution plates was added. The cell plates were incubated for 1 h at 37 °C, 5% CO_2_ in a humidified incubator. Following incubation, the sample dilutions were carefully removed and 100 µL overlay media containing 1% CMC and 1/150 volume TrypLE Select (Gibco) added to each well. Plates were incubated for 17–22 h at 37 °C, 5% CO_2_ in a humidified incubator.

Following incubation, overlay was removed, and the plates were fixed with 100% ice cold methanol for a minimum of 15 min. Plates were washed 3 times with 200 µL per well PBS, and 50 µL per well primary antibody (Mouse monoclonal anti-influenza NP at 1:1000) was added for 1 h at room temperature. Plates were again washed 3 times with 200 µL PBS, and 50 µL secondary antibody (Goat anti-mouse IgG-alkaline phosphatase at 1:1500) was added for 1 h at room temperature. Plates were washed 3 times with 200 µL PBS, and 50 µL filtered substrate (NBT/BCIP) was added until spots developed (~30 min). Plates were washed 3 times with 200 µL WFI and once dry, analysed using the CTL scanner and software (CTL, Germany).

**Animals. *Ferrets:*** Twenty-seven healthy ferrets (*Mustela putorius furo*) aged 15–19 weeks were obtained from a UK Home Office accredited supplier (Marshalls Biosciences, UK). ***Hamsters:*** Twenty-four healthy, Golden Syrian hamsters (*Mesocricetus auratus*), aged either 6 weeks (young) or 20–22 weeks (older), were obtained from a UK Home Office accredited supplier (Envigo, UK). Ferrets were housed in social groups of 3–7 animals (females) or singly (males), hamsters were housed individually at Advisory Committee on Dangerous Pathogens (ACDP) containment level 2. Cages met with the UK Home Office *Code of Practice for the Housing and Care of Animals Bred, Supplied or Used for Scientific Procedures* (December 2014). Access to food and water was ad libitum, and environmental enrichment was provided. All experimental work was conducted under the authority of a UK Home Office approved project licence that had been subject to local ethical review at UK Health Security Agency Porton Down by the Animal Welfare and Ethical Review Body (AWERB) as required by the *Home Office Animals (Scientific Procedures) Act 1986*. 

Before the start of the experiment, animals were randomly assigned to challenge groups to minimise bias. Two weeks prior to infection, a data logger (Star-Oddi, Iceland) was surgically implanted subcutaneously into the nape of the neck of each animal under anaesthesia. Loggers were programmed to take temperature readings every hour. Prior to challenge, animals were sedated by isoflurane. Virus was delivered by intranasal instillation (200 µL total, 100 µL per nostril) diluted in phosphate buffered saline (PBS).

**Clinical observations.** Ferrets and hamsters were monitored for clinical signs of disease twice daily (approximately 8 h apart). Clinical signs of disease in ferrets were recorded as follows; healthy; nasal discharge; nasal congestion/mouth breathing; nasal rattles/sneezing; laboured breathing; temperature variance (>2 °C for >48 hr) and activity level. Animals were weighed at the same time of each day from the day before infection until euthanasia. 

**Necropsy Procedures.** Ferrets and hamsters were given an anaesthetic overdose (sodium pentabarbitone Dolelethal, Vetquinol UK Ltd., 140 mg/kg) via intraperitoneal injection, and exsanguination was effected via cardiac puncture. Necropsy was performed immediately after confirmation of death.

**Haemagglutination Inhibition Assay.** Ferret and hamster sera were added to receptor-destroying enzyme (Deben Diagnostics, Ipswich, UK) in a 1:3 ratio and incubated at 37 °C for 18–20 h. The sera were then heat inactivated at 56 °C for 45 min. Treated sera were serially diluted 1:2 and added to an equal volume of virus (8HAU/well). Virus/antibody complexes were incubated at room temperature for 30–40 min before addition of one-well volume of 0.5% chicken red blood cell suspension. Virus/serum/red blood cell mix was incubated at room temperature for 30–45 min and HAI endpoints recorded as the reciprocal of the highest dilution of serum able to fully prevent agglutination.

**Neuraminidase Inhibition Assay.** NAI titres of sera were measured using the enzyme-linked lectin assay (ELLA) essentially as described by Couzens et al. [7]. The NA test material was prepared by treating GM19 virus with 1% *v*/*v* Triton X-100. Titres were expressed as the 50% inhibitory concentration determined by Sigmoidal 4PL curve fitting (GraphPad Prism).

**Ferret and Hamster PBMC Isolation.** Ferret Peripheral Blood Mononuclear Cells (PBMCs) from whole heparinised blood were isolated by density gradient centrifugation using Histopaque (Sigma). Hamster PBMCs were isolated by diluting whole blood 1:2 with HBSS (Gibco) and centrifuging at 400× *g* for 5 min. The plasma layer was discarded and 10 mL ACK cell lysis buffer added to the cell pack volume. The cell suspension was gently rocked for 10 min at RT before centrifuging at 400× *g* for 5 min. The supernatant was discarded and the pellet resuspended in 40 mL PBS before repeating the centrifugation step. PBMCs were resuspended in culture media (RPMI 1640 advanced, supplemented with 5% FCS, 1% L-glutamine and 1% antibiotic/antimycotic, 25 mM HEPES), and the cells were counted using a NucleoCounter® NC-200™ (ChemoMetec). 

**Ferret and Hamster Lung MNC Isolation.** Lung MNC isolation was performed as previously described [8].

**Hamster Splenocyte MNC Isolation.** Splenocytes were isolated from the spleen on the day of necropsy. Excess fat was trimmed from each spleen before being placed into a 50 mL centrifuge tube containing 5 mL of digestion mix (10 mM HEPES, 20 µg/mL DNAse I, 2 mg/mL Collagenase, 1x HBSS). The spleens were cut into ~2 mm^2^ pieces and incubated for 20 min at 37 °C + 5% CO_2_ using a MACSmix^TM^ Tube Rotator. The digested tissue was pressed through a 100 µm cell strainer using the flat side of a syringe plunger and the strainer rinsed with 10 mL wash media (RPMI 1640 advanced, supplemented with 1% FCS, 1% L-glutamine and 1% antibiotic/antimycotic, 25 mM HEPES and 0.5 mM EDTA). The cells were centrifuged for 5 min at 400× *g* and the supernatant discarded before the cells were resuspended in 2.5 mL ACK cell lysis buffer for 5 min. After this time, the ACK was diluted with PBS to a total volume of 30 mL and the cells pelleted at 400 g for 5 min. The cells were resuspended in 500 µL wash media, and any clumps that could not be resuspended were removed. Each tube was topped up to 10 mL and the centrifuge step repeated. The cell pellet was resuspended in 5 mL culture media (RPMI 1640 advanced, supplemented with 5% FCS, 1% L-glutamine and 1% antibiotic/antimycotic, 25 mM HEPES) and the cells counted using a NucleoCounter® NC-200™ (ChemoMetec). 

**ELISpot.** IFNγ ELISpot assay was used to estimate the frequency and IFN-γ production capacity of influenza-specific T cells in PBMCs, lung MNCs and splenocytes using either a Ferret or Hamster IFNγ kit (MabTech, Nacka, Sweden). Ferret PBMCs and lung MNCs were assayed at 1.25 × 10^5^ and 1 × 10^5^, respectively. Hamster cells were assayed at 3 × 10^5^ cells per well. Cells were stimulated overnight with MP1, MP2, HA, NA or NP Peptivator Peptides (Miltenyi Biotec) or wild-type H1N1 A/Guangdong-Maonan/SW1536/2019 at an MOI of 0.08. Cell stimulation cocktail (500×) (eBiosciences) was used at 1× as a positive control. Results were calculated to report as spot-forming units (SFU) per million cells. All stimulants were assayed in duplicate and media-only wells subtracted to give the antigen-specific SFU. ELISpot plates were analysed using the CTL scanner and software (CTL, Germany) and further analysis carried out using GraphPad Prism (GraphPad Software, USA). 

**Whole blood immunophenotyping.** First, 50 µL of whole blood was collected via gingival bleed into lithium heparin screw cap tubes (International Scientific Supplies). Whole blood was then incubated with staining antibody at a dilution of 1 in 20 (CD8b Monoclonal Antibody PE, Invitrogen; CD4 anti-mouse antibody-APC, Biolegend) and Live-Dead at a 1 in 10 dilution (Invitrogen) for a total of 30 min in the dark. The red blood cells were lysed and fixed by addition of 1 mL of 1× Fix/Lyse solution (Invitrogen) and incubated in the dark for a minimum of 15 min. Then, 200 µL of each sample was added to a polystyrene U-bottom 96 well plate (Thermo Scientific), and 20 µL of thoroughly mixed counting beads (Beckman Coulter) were added to each well. Flow cytometric analysis was performed using a Cytoflex flow cytometer (Beckman Coulter), and data were acquired using CytEpert software. Acquisition was set to count 10,000 events in the counting bead gate, with a stopping time of 8 min. Fluorescence spill-over was accounted for by applying a compensation matrix to each sample. Lymphocytes, counting beads, CD4+ and CD8b+ populations were gated using the CytExpert software. Population counts were then exported from CytExpert to Excel and the number of each cell type per millilitre quantified by the following calculation:

$$\left(\frac{cell\;events}{counting\;bead\;events}\times \frac{number\;of\;counting\;beads\;added}{total\;volume\;of\;sample\;run}\right)\times 1000$$

**Histopathology and immunohistochemical detection of viral nucleoprotein**. Lung and upper respiratory tract samples (nasal turbinates for both species, plus trachea and larynvx in hamsters) were fixed in 10% neutral buffered formalin. Nasal cavity samples were decalcified using an EDTA solution prior to obtaining a longitudinal tissue section, including the respiratory and olfactory mucosa before embedding in paraffin wax. Then, 4 µm thick sections were cut and stained with haematoxylin and eosin (H&E). Stained slides were scanned by a Hamamatsu NanoZoomer S360 scanner and evaluated (viewed) with NDP.view2 software. The pathologist was blinded to treatment and group details, and the slides were randomised prior to examination in order to prevent bias (blind evaluation). 

A combination of semi-quantitative, subjective scoring systems was used to evaluate the severity of lesions observed in the lung. Two different scoring systems were used; these are two well-established systems previously developed by Morgan et al. in 2016 [9] and Gauger et al. in 2012 [10] to evaluate histopathological lesions observed in pigs experimentally infected with Influenza virus. In addition, the percentage of area comprising pneumonia in the lung was calculated using digital image analysis (Nikon-NIS-Ar). The Leica Bond Rxm and polymer refine detection kit with HRP were used to visualise the influenza viral nucleoprotein (NP) by immunohistochemistry (IHC). Sections were dewaxed, rehydrated and treated in 3–4% hydrogen peroxide for 5 min to quench endogenous peroxidase activity. Anti-influenza NP monoclonal antibody (HB-65) and hybridoma culture supernatant were used at 1:3 dilution and incubated for 30 min. Leica polymer refine detection kit was used, and DAB as chromogen for visualisation and sections were counterstained with Harris’ haematoxylin. Positive control sections and negative controls were used in the IHC runs. For nasal turbinates, trachea and larynx, a semiquantitative scoring system was applied to evaluate the presence of virus NP: 0 = no positive staining; 1 = minimal; 2 = mild; 3 = moderate and 4 = abundant staining. For the lung IHC-stained sections, the scoring system described by Gauger et al. in 2012 was used.

## 3. Results

### 3.1. Study Design

Ferrets and hamsters were surgically implanted subcutaneously (in the nape of the neck) with data loggers two weeks prior to infection, enabling body temperatures to be recorded hourly. Animals were weighed daily, and clinical signs of infection were recorded twice daily. Ferrets (n = 24; 12 = male, 12 = female) were infected with two different doses of H1N1 GM19 at 1 × 10^6^ PFU (high, n = 12; 6 = male, 6 = female) and 1 × 10^2^ PFU (low, n = 12; 6 = male, 6 = female) in 200 µL. These two doses were selected to account for the standard infection dose in ferrets used in pathogenesis and preclinical evaluation studies [11,12,13,14] and our previously characterised and preferred ‘low dose’ [8,15,16,17]. In order to measure viral shedding in the upper respiratory tract, ferret nasal washes were obtained daily from days 1 to 8 and at day 14 post-infection. At day 4, ferrets (n = 6) from each dosing group were culled. Blood and lungs were taken for assessment for immune responses at day 4 post-infection. Nasal turbinates, including respiratory and olfactory mucosa, and lung were taken for assessment of viral load and pathology. The remaining ferrets (n = 6 per dose) had small volumes of blood taken at 7, 11 and 14 days post-infection to assess immune response kinetics. All remaining ferrets were culled at day 14 with blood and lungs taken for assessment for immune responses and nasal turbinates and lung taken for assessment of viral load and pathology.

Hamsters (n = 24; 12 = male, 12 = female) were grouped according to age (n = 12 ‘young’; 6 = male, 6 = female and n = 12 ‘older’; 6 = male, 6 = female) and infected with H1N1 GM19 at a dose of 1 × 10^6^ PFU in 200 µL. Only one ‘high dose’ was selected to infect hamsters due to previously published data demonstrating that a ‘high dose’ of influenza was the most successful in the golden Syrian hamster model [4,5,18,19,20,21]. Hamsters were split into ‘young’ and ‘older’ groups in order to compare the kinetics of and the response to infection at different ages. The published data have assessed influenza infection in hamsters < 10 weeks [4,5]. Using young and older hamsters here allow for the potential use of a range of ages in future work. As with ferrets, the hamster groups contained an equal male:female split. In order to measure viral shedding in the upper respiratory tract, hamsters were nasal washed at days 1, 3, 4, 5, 7, 11 and 14 post-infection. At day 4 post-infection, hamsters (n = 6) from each age group were culled. Blood, lung and spleen were taken for assessment for immune response at day 4 post-infection. Nasal turbinates, including respiratory and olfactory mucosa, and lung were taking for assessment of pathology. The remaining hamsters (n = 6 per group) had small volumes of blood taken at 1, 3, 7 and 14 days post-infection to assess immune kinetics. All remaining hamsters were culled at day 14 with blood, lungs and spleen taken for assessment for immune response, and nasal turbinates and lung taken for assessment of viral load and pathology.

### 3.2. Comparison of Disease Induced by A/Guangdong-Maonan/SW1536/2019 (GM19) 

**Figure 1:**
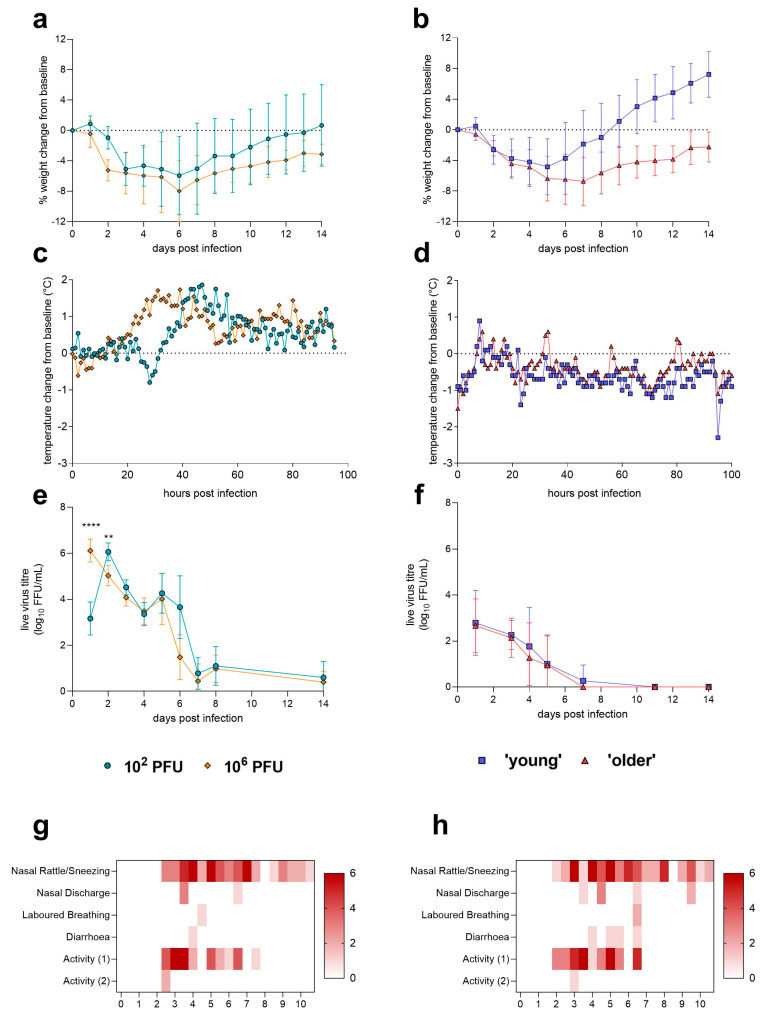
Disease kinetics of H1N1 GM19 in ferrets and Golden Syrian hamsters (**a**) Ferrets and (**b**) hamsters were weighed daily, and percentage change of body weight from baseline was calculated. Lines show mean; error bars show SD. Both (**c**) ferrets and hamsters were implanted with data loggers that measured temperature every hour. Lines show mean. (**d**) Post-infection significantly lower temperatures were recorded in young hamsters compared to older hamsters (*p* = 0.0028). Lines show mean. (**e**) Nasal washes were taken from ferrets once daily until day 8 post-infection and at day 14 post-infection. Significantly (*p* < 0.0001) higher amounts of live virus were shed by the 10^6^ PFU (high) dose-infected ferrets at day 1 post-infection. At day 2 post-infection, the 10^2^ PFU (low) dose-infected ferrets were shedding significantly (*p* = 0.0071) more live virus. Statistical analysis was performed using a mixed-effects analysis on log10-transformed data. Lines show mean; error bars show SD. (**f**) Nasal washes were taken from hamsters every other day until day 7 post-infection and at days 11 and 14 post-infection. No significant differences were observed between young and older hamsters. Lines show mean; error bars show SD. Clinical signs of infection in ferrets were recorded twice daily in the (**g**) 10^2^ PFU (low) dose and (**h**) 10^6^ PFU (high) dose-infected groups. Activity (1): playful only when stimulated, Activity (2): not playful when stimulated. Hamsters were monitored twice daily but no clinical signs were observed following infection. ** *p* < 0.01, **** *p* < 0.0001.

Both groups of ferrets experienced weight loss below baseline following infection (Figure 1a), with ferrets infected with the high dose, experiencing, on average, a trend for more weight loss. Of the ferrets that were followed to 14 days post-infection (dpi), weight loss was between 0.15 and 15.3%, with a mean of 7.6% (low dose)–7.7% (high dose). Neither dose nor sex had a significant effect on differences in weight loss. Both ages of hamsters experienced weight loss below baseline following infection (Figure 1b), with older hamsters experiencing, on average, more and sustained weight loss and mean peak weight loss to compared to ferrets. By day 10 post-infection, young hamsters were found to be gaining significantly (P 0.0365) more weight than older hamsters; however, this is likely due to their normal rate of growth and not a direct result of influenza infection. Male hamsters also experienced on average more weight loss than females (Figure S1a) with a similar trajectory of recovery following infection. Differences observed in weight loss between female and male hamsters were not significant.

Baseline temperature was calculated as the mean of all readings from three days before infection until just prior to infection. Female ferrets were found to have significantly higher baseline temperatures (*p* < 0.0001) compared to males (Figure S1d). Post-infection temperatures were expressed as difference from the baseline specific to that animal. The high-dose-infected ferrets experienced a more rapid rise in temperature compared to the low-dose-infected ferrets, with a peak at approximately 30–40 h post-infection (hpi) compared to 40–60 hpi (Figure 1c). A marked reduction in temperature was observed in the low-dose-infected ferrets between 28 and 30 hpi. The mean temperatures in high-dose-infected ferrets did not decrease to baseline until approximately 130 hpi (between 5 and 6 days post-infection). The maximum temperature rises were approximately 2 °C for both doses, and all 24 ferrets showed transient fever as a result of infection, with high-dose-infected ferrets experiencing a fever significantly (*p* < 0.0001) earlier than low-dose-infected ferrets (Figure S2a). 

Temperature monitoring of hamsters showed that both groups experienced a significant temperature drop below baseline following infection (Figure 1d). Data also show that following infection, both groups of hamsters experienced a disruption to the diurnal rhythm observed prior to infection. No differences were observed between males and females prior to infection (Figure S1b), but young hamsters showed lower temperatures than older hamsters (*p* = 0.0028), and male hamsters showed lower temperatures than female hamsters (*p* = 0.0004) post-infection. All ferrets exhibited clinical signs following infection, with sneezing and inactivity being the most frequently observed signs (Figure 1g,h). There was a trend for more frequent observations in the high-dose group; however, this was not significant. For both doses, clinical sign onset was from approximately 2 days post-infection, continuing until approximately 11 days post-infection. Hamsters were monitored for clinical signs of influenza infection throughout; none were noted. 

Live virus shedding in the upper respiratory tract was measured in ferret nasal washes collected at days 1–8 and 14 post-infection (Figure 1e). Upper respiratory tract shedding in high-dose ferrets peaked at one dpi (9/12 ferrets) and was found to be significantly higher (*p* < 0.0001) than virus shed in the low-dose group. Virus shedding peaked at two dpi (12/12 ferrets) in the low-dose-infected ferrets and conversely was found to be significantly higher (*p* = 0.0071) than virus shed in the high-dose ferrets at the same time point. No clear difference in shedding was found between males and females. There was no significant difference in peak titres between high dose (mean 1.6 × 10^6^ FFU/mL) and low dose (mean 1.3 × 10^6^ FFU/mL). A distinctive ‘second peak’ of viral shedding was observed in both the high-dose and low-dose ferrets at 5 days post-infection. This has been noted previously in the ferret model by these authors [8] and others [22]. Viral load in the lung and nasal turbinates was assessed at 4 and 14 days post-infection for high- and low-dose ferrets (Figure S2b). The viral load in low-dose ferrets at day 4 post-infection was observed to be lower (mean 3.28 logs) than high-dose ferrets (mean 4.63 logs) at the same timepoint, but this was not significant. The viral loads in the nasal turbinates of low-dose and high-dose-infected ferrets were very similar at day 4 post-infection (mean 6.28 vs. 5.91 logs). Females had a significantly lower viral titre than males (*p* = 0.0108) found in nasal turbinates at day 4 post-infection regardless of dose (Figure S1e). Live virus was undetectable in tissues by day 14 post-infection for most ferrets regardless of dose received, mirroring upper respiratory tract shedding observed at day 14 post-infection.

Live virus shedding in the upper respiratory tract of hamsters was measured in nasal washes collected at days 1, 3, 4, 5, 7, 11 and 14 post-infection (Figure 1f). Upper respiratory tract shedding in the majority of hamsters peaked at 1 dpi (mean 7 × 10^3^ FFU/mL in young and 2 × 10^3^ in older hamsters), regardless of age, and a trend for younger hamsters to shed more live virus in their respiratory tract was observed; however, this was not significant. A significant difference in shedding was found between males and females at day 1 post-infection (*p* = 0.0012), and a trend for higher levels of shedding in male hamsters continued until 5 days post-infection (Figure S1c). All male hamsters had recoverable live virus in nasal washes with at least two timepoints measured, while 2/12 female hamsters had no recoverable live virus at any time point. Viral load in the lung was assessed at 4 and 14 days post-infection for young and older hamsters (Figure S2c). Due to an assay failure, only two reliable results of detectable viral load were detected in two young male hamsters at 4 days post-infection.

**Figure 2:**
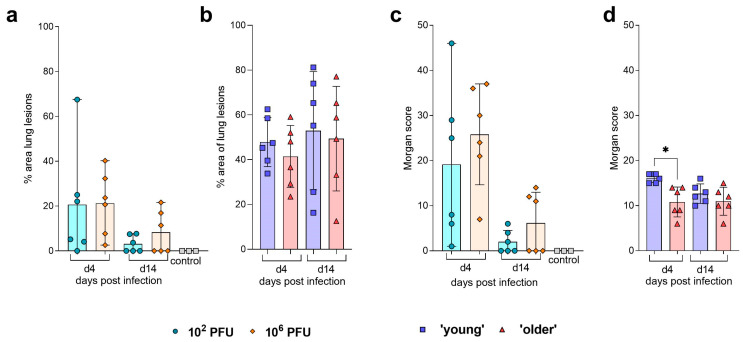
Pathology in ferrets and Golden Syrian hamsters following infection with H1N1 GM19 (**a**) Analysis of percentage area of pneumonia in the lung was carried out at days 4 and 14 post-infection and compared to control ferrets. Symbols show individual ferrets, lines show mean, and error bars show SD. (**b**) At days 4 and 14 post-infection, analysis of percentage area of pneumonia in the lung of hamsters was carried out. Lines show mean, error bars show SD. (**c**) Scoring using the Morgan scoring system [9] was assigned to each ferret at days 4 and 14 post-infection and compared to control ferrets. Symbols show individual ferrets, lines show mean, error bars show SD. (**d**) Scoring using the Morgan scoring system [9] was assigned to each hamster at days 4 and 14 post-infection. Young hamsters were assigned a significantly higher score at day 4 compared to older hamsters. Statistical analysis was performed using Kruskal–Wallis. Lines show mean; error bars show SD, * *p* <0.05.

Histopathological examination of ferret nasal turbinate sections at day 4 post-infection showed multifocal necrotising rhinitis, with cell death observed in epithelial and sustentacular cells within the respiratory and olfactory mucosa. Mixed inflammatory cell exudates were also observed within the nasal cavity. In the lung, severe multifocal broncho-interstitial pneumonia was observed, with necrosis and attenuation of the bronchiolar and bronchial epithelium, suppurative infiltrates within the bronchiolar and bronchial lumina and the alveolar spaces together with alveolar septal inflammatory cell infiltration (Figure S3a). Moreover, perivascular lymphoplasmacytic cuffing was frequently observed. Virus nucleoprotein antigen (NP) was observed by immunohistochemistry (IHC), mainly within the respiratory and olfactory mucosa epithelial and sustentacular cells in the nasal turbinates (Figure S3c), and the bronchiolar epithelium and inflammatory infiltrates within the parenchyma and the airways in the lung (Figure S3b). The severity of histopathological lesions and presence of virus NP was evaluated using a scoring system used in experimental porcine influenza [23,24], combining two scoring systems previously described [9,10]. A reduction in the area of lung lesions was observed between days 4 and 14 post-infection in both doses (Figure 2a). This is mirrored in the mean Morgan score (Figure 2c), which is higher at day 4 post-infection for both high- and low-dose-infected ferrets with a reduced score seen at day 14 for both. No virus NP was detected by IHC in the nasal turbinates or lung at day 14 post-infection.

Histopathological lesions consistent with infection with influenza virus infection were observed in the lungs and nasal cavity in all the infected hamsters (Figure S4). Lesions at day 4 post-infection in the upper respiratory tract were consistent in mild necrosis and attenuation of the epithelium together with inflammatory reaction in the nasal epithelium and were only minimal (if present) in the trachea and larynx. Lesions and presence of abundant virus NP by IHC were observed in both the respiratory and olfactory mucosa at day 4 post-infection (Figure S4c). Only a small amount of virus NP was detected occasionally in the trachea and larynx of some animals, associated with minimal lesions within the epithelium. At day 14 post-infection, neither lesions nor presence of virus NP were observed in the upper respiratory tract.

Pulmonary lesions at day 4 post-infection consisted in acute bronchopneumonia with necrosis and attenuation of airway epithelial cells, inflammatory cell infiltration within the airways, and alveoli and septa and perivascular cuffing (Figure S4a). In the lung, IHC detection of the virus NP was observed at the same timepoint, mainly in airway epithelial cells and within inflammatory infiltrates in the alveoli and septae (Figure S4b). At day 14 post-infection, lesions were less severe and more subacute, and no virus NP was observed in any hamster. No differences were observed in the area of lung lesions between days 4 and 14 post-infection in both groups (Figure 2b). A significantly higher (*p* = 0.0125) pulmonary histopathology score (‘Morgan’) was seen in younger hamsters at day 4 post-infection compared to older hamsters. This trend continued to day 14 post-infection but was not found to be significant (Figure 2d). 

### 3.3. Comparison of the Host Response Induced by A/Guangdong-Maonan/SW1536/2019 (GM19) Infection

**Figure 3:**
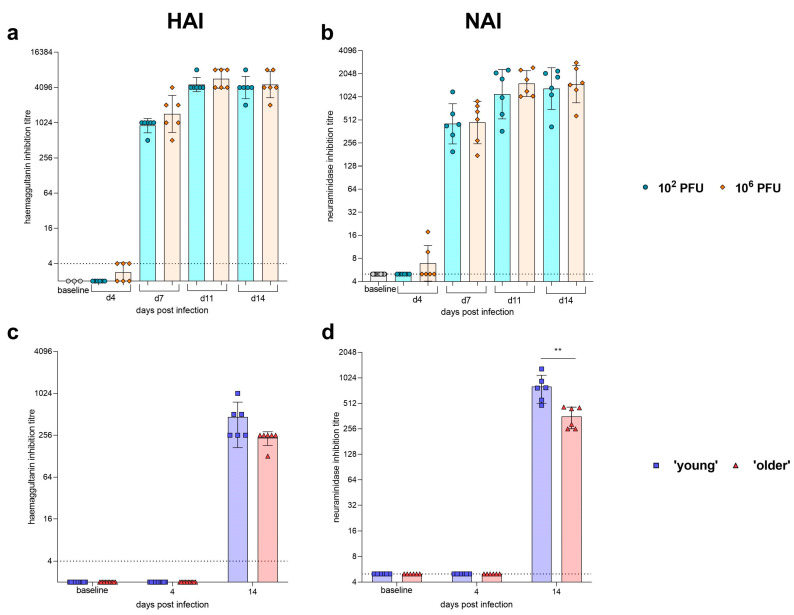
**Humoral response to H1N1 GM19 influenza infection in ferrets and the Golden Syrian hamster.** Sera were collected from ferrets at baseline, days 4, 7, 11 and 14 post-infection; (**a**) haemagglutination inhibition and (**b**) neuraminidase inhibition were assessed. All ferrets, irrespective of dose received, seroconverted by 7-day post-infection. No differences were observed between doses. Sera were collected from hamsters at baseline and days 4 and 14 post-infection; (**c**) haemagglutination inhibition and (**d**) neuraminidase inhibition were assessed. All hamsters irrespective of dose received seroconverted by 14 days post-infection. A significant difference at day 14 post-infection was observed between young and older hamsters for neuraminidase inhibition (** *p* = 0.0028, unpaired *t*-test).

Ferret sera were assessed for the ability of antibodies to inhibit agglutination of chicken red blood cells by GM19 virus (Figure 3a). All ferrets were pre-screened against GM19 and were found to be seronegative (titres < 4). At 4 days post-infection, titres were not significantly above baseline. A significant increase was observed between days 4 and 7, and days 7 and 11 post-infection regardless of dose received. There was a trend observed of higher titres in the high-dose group at day 7; however, this was not significant. HAI titres reached a maximum at day 11 post-infection, with no further increase at 14 dpi. Terminal day 14 titres were in the range 2048–8192. No differences were seen between male and female ferrets. Sera were also assessed for neuraminidase inhibition titres (Figure 3b). The NA antibody kinetics observed were similar to those observed in the HAI, with NAI titres reaching a maximum at day 11 post-infection for both high- and low-dose-infected ferrets. As with HAI, there were no differences observed between male and female ferrets. 

Hamster sera were assessed for the ability of antibodies to inhibit agglutination of chicken red blood cells by the GM19 virus (Figure 3c). All hamsters were pre-screened against GM19 and were found to be seronegative (titres < 4). At four days post-infection, titres remained at baseline. A significant increase was observed between days 4 and 14 regardless of age. There was a trend observed of higher titres in the younger hamsters; however, these were not significant. The day-14 hamster HAI titres were all lower than the ferret day-14 HAI titres. Sera were also assessed for neuraminidase inhibition titres (Figure 3d). The NA antibody kinetics observed were similar to those observed in the HAI with NAI titres, with a significant increase at day 14 post-infection for both young and older hamsters and with younger hamsters having significantly (P = 0.0028) higher titres. No differences in seroconversion were observed between male and female hamsters. The hamster NAI titres were comparable to the ferret NAI titres at day 14.

**Figure 4:**
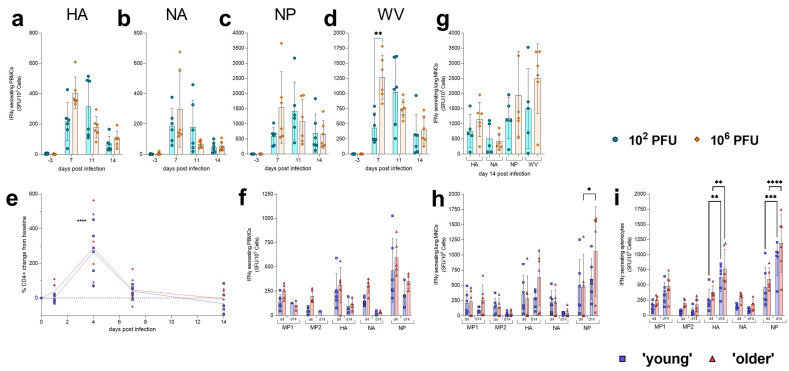
Cellular response to H1N1 GM19 influenza infection in the ferret and Golden Syrian hamster. PBMCs were collected at baseline and days 7, 11 and 14 post-infection to assess longitudinal IFNγ responses. PBMCs were stimulated with (**a**) HA, (**b**) NA, (**c**) NP and (**d**) whole virus (GM19). A significant (** *p* = 0.0052) difference between the 10^6^ PFU (high)-dose-infected ferrets and the 10^2^ PFU (low)-dose-infected ferrets was detected at day 7 post-infection when PBMCs were stimulated with homologous virus. Statistical analysis was performed used mixed-effects analysis. (**e**) Small blood volumes were collected from the gingiva of hamsters at baseline and days 1, 4, 7 and 14 post-infection. The percentage change of CD4+ cells from baseline was calculated, and a significant (**** *p* < 0.0001) increase in cells was observed in both ages of hamsters. Statistical analysis was performed using mixed-effects analysis. Symbols show individual hamsters; lines show means. (**f**) PBMCs were collected at cull to assess IFNγ responses. PBMCs were isolated from hamsters at days 4 and 14 post-infection and were stimulated with M1, M2, HA, NA, and NP. (**g**) At 14 days post-infection, ferret lung MNCs were assessed for influenza-specific IFNγ responses. Bars show mean; error bars show SD. (**h**) Hamster lung MNCs * *p* < 0.05 and (**i**) splenocytes were isolated at days 4 and 14 post-infection and were stimulated with M1, M2, HA, NA, and NP. A significant increase from day 4 to 14 in HA- (** *p* < 0.01) and NP- (*** *p* = 0.0005, **** *p* < 0.0001) specific IFNγ responses in both young and older hamsters, respectively, was observed in splenocytes. A significant increase from day 4 to 14 in NP- (* *p* = 0.0112) specific IFNγ responses in older hamsters was also observed in lung MNCs. Statistical analyses were performed using two-way ANOVA. Bars show mean; error bars show SD.

Ferret cellular immune responses to infection were measured in circulating whole blood (PBMCs) and in the lung (lung MNCs). Cells were stimulated with either peptide pools (HA, NA or NP derived from H1N1 A/Cal/07/09) or homologous whole virus (GM19). Influenza-specific IFNγ responses to infection were then analysed using ELISpot. Sequential small bleeds allowed for longitudinal influenza-specific IFNγ responses in the ferrets to be monitored over 14 days. Influenza-specific IFNγ responses in peripheral circulating blood showed a trend for ferrets infected with the high dose to have higher responses at day 7 post-infection for HA (Figure 4a), NA (Figure 4b), NP (Figure 4c) and homologous whole virus (Figure 4d) compared to those infected with the low dose, but these were only found to be significant against whole virus (*p* 0.0052). By day 11 post-infection, these responses were higher in the low-dose-infected ferrets. In general, the highest responses, regardless of dose, were seen for nucleoprotein (NP). Influenza-specific IFNγ responses in lung MNCs were assessed in ferrets at day 14 post-infection (Figure 4g). On average, IFNγ responses observed against HA, NP and wild-type homologous virus were highest in the lung low-dose-infected ferrets on day 14; however, these differences were not found to be significant. 

Hamster cellular responses to infection were measured in circulating whole blood by sequential sampling at days 1, 4, 7 and 14 post-infection (Figure 4e). An increase in CD4+ lymphocytes was observed in both groups at day 4 post-infection, including a significant increase (*p* < 0.0001 for both younger and older hamsters) from levels of circulating CD4+ lymphocytes at day 1 post-infection. By day 7 post-infection, these levels had significantly (*p* = 0.0002, <0.0001 respectively) dropped towards baseline levels from a day 4 peak. At days 4 and 14 post-infection, whole blood (PBMCs), lung (lung MNCs) and spleen (splenocytes) were collected to assess influenza-specific IFNγ responses. Cells were stimulated with peptide pools (MP1, MP2, HA, NA or NP) derived from H1N1 A/Cal/07/09. Influenza-specific IFNγ responses to infection were then analysed using ELISpot. Influenza-specific IFNγ responses in terminal blood (Figure 4f) showed a trend for infected hamsters to have higher responses at day 4 post-infection to MP1, MP2, HA, NA and NP compared to day 14 post-infection in both young and older hamsters. In general, the highest responses, regardless of age, were seen for nucleoprotein (NP). Influenza-specific IFNγ responses in lung MNCs were assessed in hamsters at days 4 and 14 post-infection (Figure 4h). On average, IFNγ responses observed against MP1, MP2, HA and NA were similar across both days and groups, with no specific trends. IFNγ responses to NP were found to be significantly (*p* = 0.0112) higher at day 14 post-infection compared to day 4 post-infection in older hamsters. This increase was not significant in younger hamsters. Splenocytes were also assessed for influenza-specific IFNγ responses (Figure 4i). In general, responses detected in the spleen were higher for both groups at 14 days post-infection compared to 4 days post-infection. These responses were found to be significantly higher against HA (*p* = 0.0092, 0.0086) and NP (*p* = 0.0005, <0.0001) in both young and older hamsters respectively.

## 4. Discussion 

In these studies, we confirmed that Golden Syrian hamsters are susceptible to influenza H1N1 GM19, a contemporary human seasonal H1N1, and we demonstrated how the disease progression, viral kinetics and host immune response compared to ferrets (Table 1), the gold standard model for influenza, infected with the same strain. We compared a high-dose and low-dose (previously characterised [8,15,16,25]) infection in ferrets to a high-dose infection in hamsters of two different ages. Our decision to test only 1 × 10^6^ PFU of influenza in the hamsters was based on previously published data [4,5,18,19,20,21] in Golden Syrian hamsters. 

Here, we reported similar weight loss below baseline following infection for both ferrets and hamsters, with clinical signs of infection observed in ferrets but not in hamsters. High-dose-infected ferrets saw significantly more weight loss at day two post-infection compared to low-dose-infected ferrets; however, no differences in the frequency or type of clinical sign recorded were observed. Both groups of hamsters lost weight following infection, and a significant difference was observed between ‘young’ and ‘old’ hamsters in weight loss, with old hamsters failing to gain weight as quickly as the younger hamsters. This is likely due to the speed at which younger hamsters gain weight and not a direct cause of influenza infection. Hamsters showed no observable clinical signs of infection in line with previous reports [4,5]. All ferrets were recorded experiencing a fever following infection with influenza. Temperature analysis in ferrets shows the peak of fever occurring significantly sooner after infection in ferrets receiving a high dose. Hamsters did not experience a fever; conversely, both groups saw a drop in temperature below baseline and a disruption of the regular diurnal cycle following infection. This temperature drop was found to be significantly lower in male hamsters. A similar drop in temperature was observed in hamsters infected with SARS-CoV-2 [26].

We were able to observe live virus shedding in the upper respiratory tract of both ferrets and hamsters. Differences in the peak of live viral shedding were observed in high- (peak at day 1 post-infection) and low- (peak at day two post-infection) dose-infected ferrets, with a previously observed ‘double peak’ observed at both doses. No differences were observed between ‘young’ and ‘old’ hamsters in shedding, and our findings are in line with other reports that, after day six post-infection, shedding of live virus ceases. However, we observed a significant difference between shedding in male and female hamsters at day 1 post-infection. We also found differences in sex when comparing the disease outcomes of influenza infection in Golden Syrian hamsters. Male hamsters appeared to fair worse with a trend of increased weight loss from baseline and a significant drop in temperature post-infection compared to females. This observation is similar to reports of male hamsters having a more severe infection with SARS-CoV-2 [27,28] but has not yet been described in the hamster model of influenza. Pathology was found to be similar in both species, with severe bronchopneumonia observed at four days post-infection in both ferrets and hamsters. This suggests that even though the viral load in the lung in hamsters was not ascertained due to an assay failure, that virus progressed to the lung. By day 14 post-infection, pathological analysis demonstrates recovery in both ferrets and hamsters. The presence of viral nucleoprotein was observed in the same cell populations in both species, similar to what is observed in pigs (and humans) [23,24].

We also characterised the humoral and cellular immune response to influenza in ferrets and hamsters, demonstrating that robust humoral responses are detected by 14 days post-infection in both ferrets and hamsters regardless of dose received (ferrets) or age (hamsters). While the humoral immune response to H1N1 GM19 is not dose dependent in ferrets, it appears that the kinetics of the cellular response is dose dependent with more rapid kinetics in the periphery observed in high-dose-infected ferrets. We found that in high-dose-infected ferrets, a peak in influenza-specific IFNγ responses was seen in peripheral blood at day seven post-infection, with a delayed peak in the low-dose-infected group seen at day 11. Robust responses were observed in ferret lungs at 14 days post-infection, with the highest response observed in the high-dose group, in line with the observation of higher viral titres observed in the lung at day 4 post-infection. The data shown here in the low-dose-infected ferrets align with our previous longitudinal assessment of influenza-specific IFNγ responses both in the periphery [16] and in the lung [8].

Here, we established that we could characterise the longitudinal cellular immune response to infection in hamsters with small blood volumes taken from the gingiva. The significant increase in CD4+ cells from baseline to day 4 post-infection in both the ‘young’ and ‘older’ hamsters is likely caused by lymphocytes moving towards the site of infection in the upper respiratory tract. The number of CD4+ cells starts to fall to baseline levels by day seven, returning to baseline levels by day 14. Influenza-specific IFNγ responses observed in peripheral blood showed that responses were higher at day 4 post-infection compared to day 14. Robust cellular responses were detected in the spleen and lung at day 14 post-infection in both the ‘young’ and ‘older’ hamsters. 

We have shown that, regardless of age, Golden Syrian hamsters have similar host immune response, disease kinetics and pathology to ferrets, also aligning with pathology and host immune kinetics observed in pigs when infected with influenza. However, unlike mice, all of these species are limited in their immunological reagent repertoire to assess the host immune response. In addition, pigs and, to a lesser extent, ferrets have logistical challenges, as limited laboratories have the expertise to work with and house them. In contrast, Golden Syrian hamsters are smaller and easier to house and handle. This is especially important when evaluating influenza strains that require CL3/BSL3 or SAPO4 containment such as H7N9 or H5N1 [18], where space is often constricted and where using larger numbers of smaller animals could provide more power for pathogenicity or protection studies.

These data demonstrate that Golden Syrian hamsters provide a promising model for preclinical assessment of influenza interventions. Hamsters have been shown to express both α2,6- and α2,3-linked sialic acid on epithelial cells in the pharynx, trachea and bronchus [29], with only α2,3-linked sialic acids expressed on the epithelial cells of the lung [4], a similar distribution to that of humans [30,31]. The possession of both α2,6- and α2,3-linked sialic acid receptors means that hamsters are a suitable model for comparative studies between viruses with different sialic acid specificities, for example, human and avian viruses. Hamsters have been used extensively over the past three years in SARS-CoV-2 studies to assess the pathogenesis of emerging variants [32,33,34], the effect of countermeasures [35,36,37] and the impact of co-infection with influenza [38] or adenovirus [39]. 

Going forward, studies to compare disease endpoints identified here in untreated and treated animals will be required to confirm that the model has sufficient discriminatory power. Although further development of the Golden Syrian hamster model of influenza infection is required, we believe that these data enhance the relevance of the model to study influenza pathogenesis, acquired immunity and vaccination strategies to influenza.

## Figures and Tables

**Table 1:** Comparison of the ferret and Golden Syrian hamster models.

	Ferret (*Mustela putorius furo*)	Golden Syrian hamster (*Mesocricetus auratus*)	
**Age**	15–19 weeks	5 weeks (‘young’)	>20 weeks (‘older’)
**Infection Dose**	1 × 10^6^ PFU in 200 μL (‘high’) and 1 × 10^2^ PFU in 200 μL (‘low’)	1 × 10^6^ PFU in 200 μL	
**Weight loss**	Yes, average peak – 6 dpi	Yes, average peak – 5 dpi (young) or 7 dpi (older)	
**Clinical signs**	Nasal rattles/sneezing, nasal discharge, laboured breathing, diarrhoea, reduced activity. No differences observed between doses.	None	
**Temperature**	Fever peak at ~35–45 hpi, depending on dose.	Temperature drop observed following infection as well as disruption to diurnal rhythm. Differences observed between male and female hamsters—male hamster temperatures fell significantly following infection.	
**Shedding of live virus from the URT**	Yes, peak observed at 1–2 dpi depending on dose, shedding was detectable until 8 dpi.	Yes, peak observed mainly at 1 dpi; shedding detected until 5 dpi. Differences observed between male and female hamsters at 1 dpi—male hamsters shedding significantly more live virus.	
**Viral load in LRT**	Live virus detected in lung at 4 dpi. Virus only detected in one (high dose) animal at 14 dpi.	Live virus detected in lung of 2/12 (male) at 4 dpi. Assay failure suspected. Method requires further optimisation.	
**Pathology**	No differences observed in % lesions in the lung. No significant differences observed in assigned ‘Morgan score’ [9] at 4 or 14 dpi. Viral NP staining was detectable at 4 dpi in the NT and lung.	No differences observed in % lesions in the lung. ‘Young’ hamsters were assigned a significantly higher ‘Morgan score’ [9] at 4 dpi. Viral NP staining was detectable at 4 dpi in the NT and lung.	
**Humoral Response**	Seroconversion detected by HAI and NAI at 7 dpi. No difference between dose observed in HAI and NAI titres.	Seroconversion detected by HAI and NAI at 14 dpi. No differences between ages observed in HAI titres. Young hamsters had significantly higher NAI titres at 14 dpi.	
**Cellular Response**	Peak of response in PBMCs observed at 7 dpi (high dose) or 11 dpi (low dose). Higher influenza-specific IFNγ responses observed in the lung of high-dose ferrets when compared to low-dose ferrets. NP-specific IFNγ responses were highest in both compartments measured.	No differences observed between ‘young’ and ‘older’ hamsters in amount of CD4+ cells in peripheral blood following infection. No differences in influenza-specific IFNγ responses observed between ‘young’ or ‘older’ hamsters. In general, higher responses were observed at 14 dpi when compared to 4 dpi. NP-specific IFNγ responses were highest in all compartments measured.	

## Data Availability

Not applicable.

## Supplementary Materials

The following supporting information can be downloaded at: https://www.mdpi.com/article/10.3390/pathogens12050668/s1, Figure S1: GM19 disease kinetics in male and females; Figure S2: Fever peak and live virus detected in tissues; Figure S3: GM19 Ferret Pathology; Figure S4: GM19 Hamster Pathology.
