# Supplementary material for: Infection with Seasonal H1N1 Influenza Results in Comparable Disease Kinetics and Host Immune Responses in Ferrets and Golden Syrian Hamsters
Source: Pathogens. 2023 Apr 30;12(5):668. doi: 10.3390/pathogens12050668 (PMC10222994; doi:10.3390/pathogens12050668)
Supplement: Supplementary file 1 [file pathogens-12-00668-s001.zip › pathogens-2301741-supplementary.pdf]

Supplementary Figures

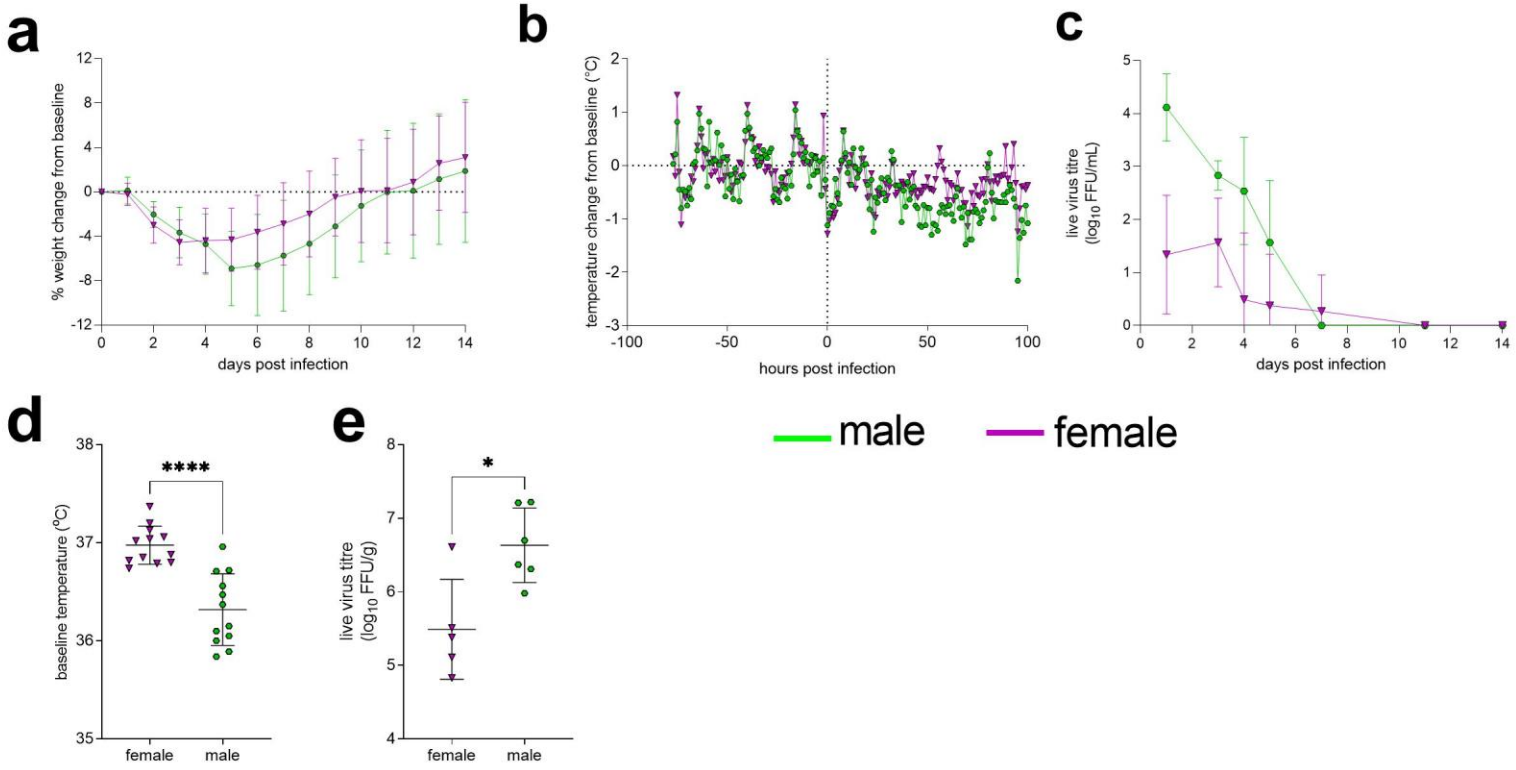

Supplementary Figure S1 GM19 disease kinetics in male and females. (**a**) Percentage weight change in hamsters from baseline. Hamsters were implanted with data loggers that measured temperature every hour. (**b**) Baseline temperature analysis showed no difference between female and male hamsters. Post infection male hamsters had lower temperatures than female hamsters (P=0.0004). Lines show mean. (**c**) Nasal washes were taken every other day until day 7 post infection and at day 11 and day 14 post infection. A significant (P=0.0012, P=0.0499) difference was observed between male and female hamsters day one and two post infection respectively. Lines show mean, error bars show 95% CI. (**d**) Baseline temperature analysis showed that female ferrets have significantly (P <0.0001) higher temperatures than male ferrets. Statistical analysis was performed using an unpaired T test. Symbols show individual ferrets, lines show mean, error bars show SD. (e) Comparison of male and female ferrets showed significantly (P=0.0108) higher viral load in the nasal turbinates of male ferrets at day 4 post infection compared to female ferrets. Statistical analysis was performed using an unpaired T test on log10 transformed data.

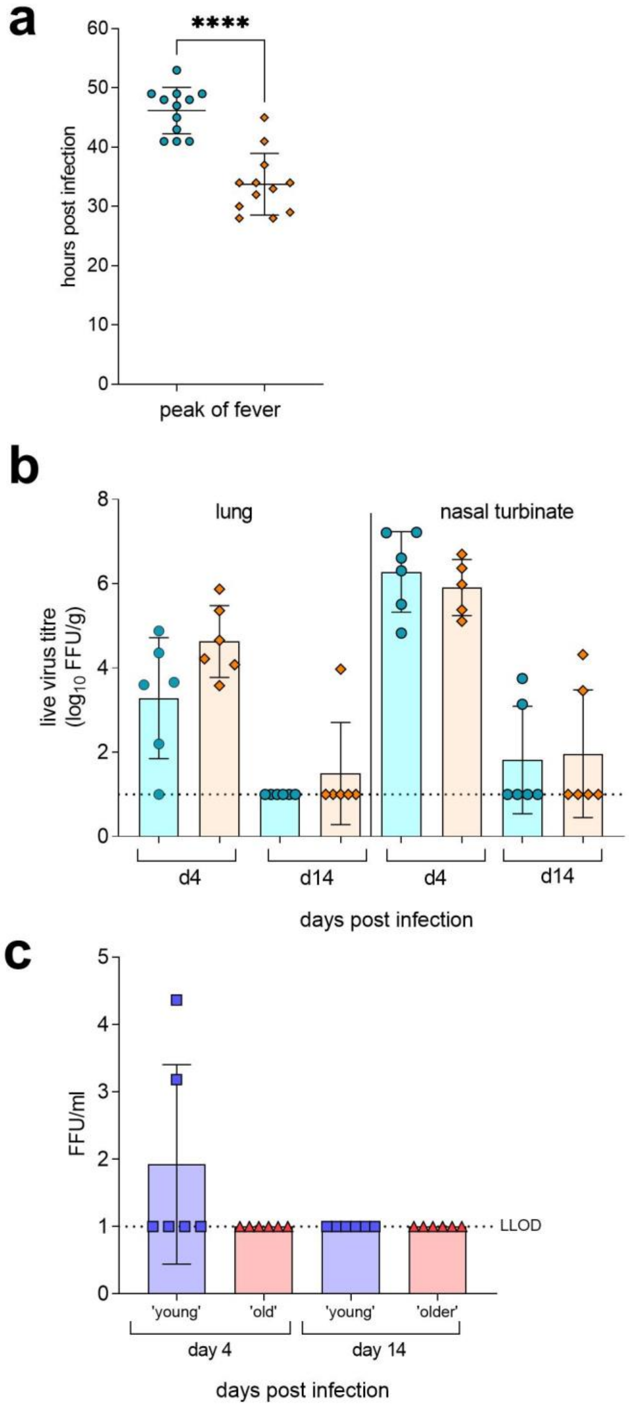

Supplementary Figure S2 Fever peak and live virus detected in tissues. (**a**) Post infection the 106 PFU (high) dose infected ferrets experienced a fever sooner than the 102 PFU (low) dose infected ferrets. Lines show mean. This was shown to be significantly earlier (P<0.0001). Statistical analysis was performed using an unpaired T test. Symbols show individual ferrets, lines show mean, error bars show SD. (**b**) At day 4 and day 14 post infection lung and nasal turbinate were taken to assess viral load. Symbols show individual ferrets, lines show mean, error bars show SD. (**d**) At day 4 and day 14 post infection lung was taken from hamsters to assess viral load. Symbols show individual ferrets, lines show mean, error bars show SD. Values at the LLOD represent an assay failure where results were unable to be ascertained. Both symbols that show positive results represent male hamsters.

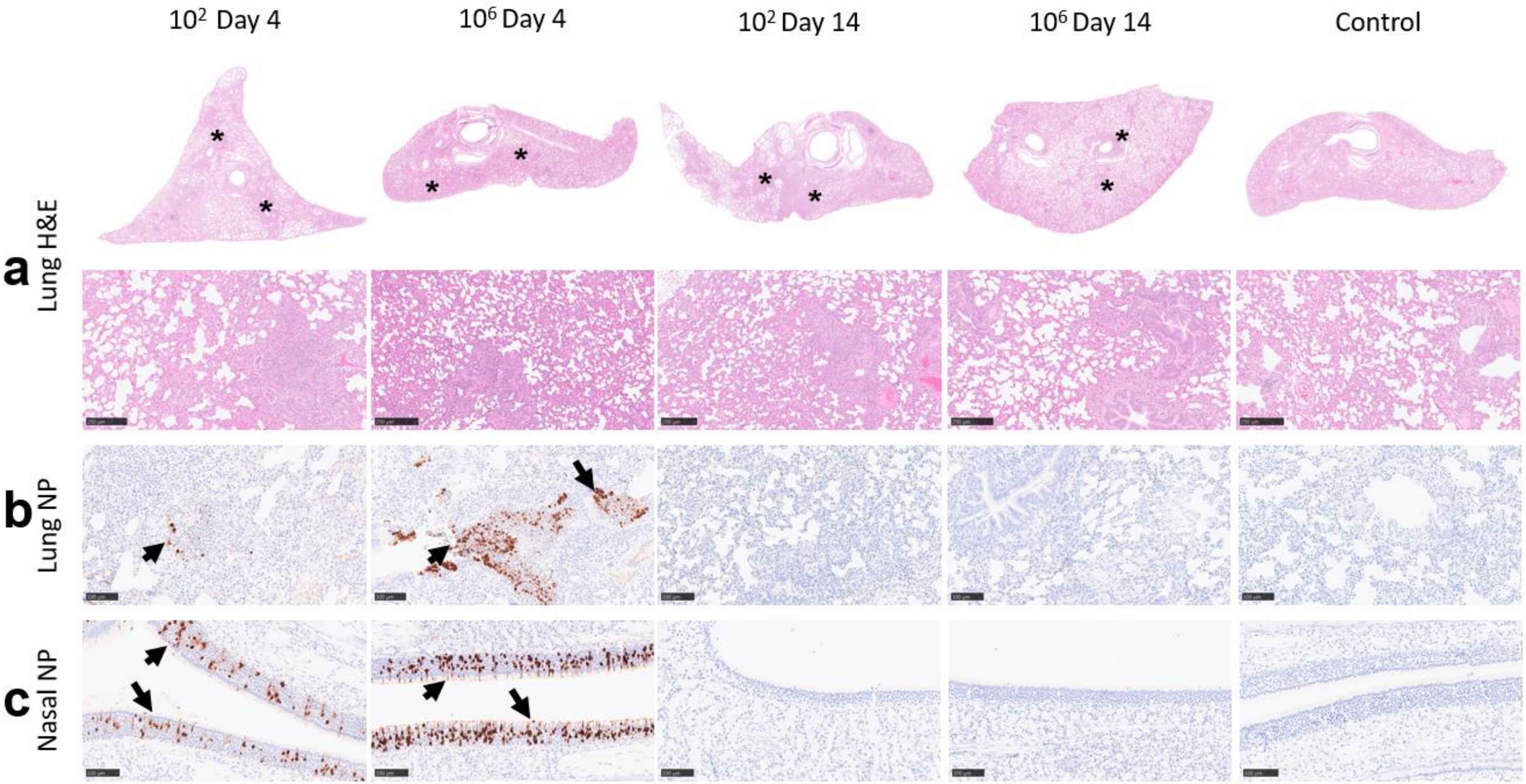

Supplementary Figure S3 GM19 Ferret Pathology. Submacro and high-power H&E images (**a**) (bar=250 µm) from the lung of ferrets culled at days 4 and 14 post influenza infection. Histopathological lesions can be observed in all infected groups (*). Immunohistochemical detection of viral nucleoprotein (NP) in the lung (**b**) (bar=100 µm) and the nasal turbinates (**c**) (bar=100 µm). Positive viral NP staining can be observed at day 4 post infection in bronchiolar epithelial cells and inflammatory infiltrates in the lung and within the nasal mucosa (arrows). No viral NP staining is observed at Day 14 or control animals.

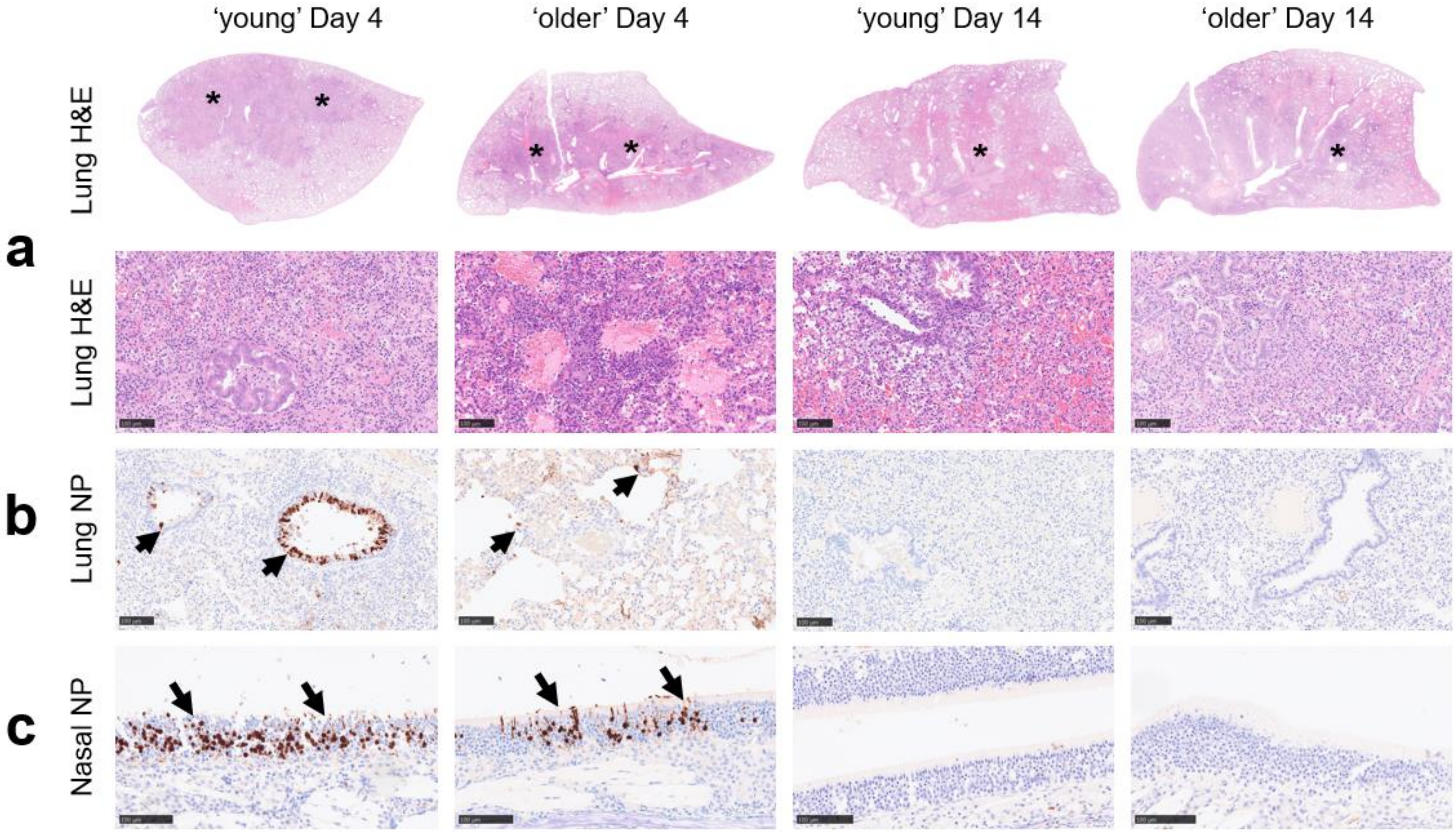

Supplementary Figure S4 GM19 Hamster Pathology. Submacro and high-power H&E images (**a**) (bar=100 µm) from the lung of hamsters culled at days 4 and 14 post infection. Histopathological lesions can be observed in all infected groups (*). Immunohistochemical detection of viral nucleoprotein (NP) in the lung (b) (bar=100 µm) and the nasal turbinates (c) (bar=100 µm). Positive viral NP staining can be observed at day 4 post infection in bronchiolar epithelial cells and inflammatory infiltrates in the lung and within the nasal mucosa (arrows). No viral NP staining is observed at day 14 post infection.
